# Advances in electrochemical immunosensors for tuberculosis detection: a systematic review

**DOI:** 10.1016/j.btre.2026.e00967

**Published:** 2026-06-15

**Authors:** Sasan Radfar, Mostafa Teymuri, Michael Holzinger, Shima Mahmoudi

**Affiliations:** a Centre for Organic and Nanohybrid Electronics, Silesian University of Technology, Konarskiego 22B, 44-100, Gliwice, Poland; b Joint Doctoral School, Silesian University of Technology, Akademicka 2A, 44-100, Gliwice, Poland; c Biotechnology Centre, Silesian University of Technology, Bolesława Krzywoustego 8, 44-100, Gliwice, Poland; d University Grenoble Alpes-CNRS, DCM, UMR, 5250, Grenoble, France

**Keywords:** Tuberculosis, *Mycobacterium tuberculosis*, Electrochemical immunosensor, Nanomaterials, Biosensors

## Abstract

Tuberculosis (TB) remains a major global health challenge, emphasizing the need for rapid and reliable diagnostic tools. As technologies move toward simplicity, portability, and real-time analysis, electrochemical biosensors have emerged as promising solutions for effective and sustainable TB detection. This systematic review highlights recent progress in electrochemical immunosensors for *Mycobacterium tuberculosis* (Mtb). A comprehensive search of PubMed, Scopus, and Web of Science identified 19 relevant studies targeting antigens such as Ag85, ESAT-6, CFP-10, MPT64, LAM, and Hsp16, with one study detecting anti-LAM antibodies. Electrode modifiers were categorized into metallic nanoparticles, conductive polymers, carbon-based nanocomposites, and ternary hybrids. Although carbon-based nanomaterials offer excellent conductivity and biocompatibility, their use in TB immunosensors remains limited. A growing trend toward label-free formats reflects the need for point-of-care compatibility. Advances in nanomaterials have improved sensor performance, but further research using next-generation modifiers and nanozymes is needed to enhance sensitivity and reproducibility.

## Highlights

• Electrochemical immunosensors are emerging as rapid, portable, and sustainable tools for TB detection, addressing the ongoing global need for reliable diagnostics.
• Nineteen studies were identified, targeting key *Mycobacterium tuberculosis* antigens such as Ag85, ESAT-6, CFP-10, MPT64, LAM, and Hsp16.
• Electrode modifiers used in these sensors fall into three main categories: metallic nanoparticles, conductive polymers, and carbon-based nanocomposites.
• Carbon-based nanomaterials remain underutilized, despite their high conductivity, large surface area, and excellent biocompatibility.
• A strong shift toward label-free detection formats is evident, but further integration of advanced nanomaterials, functional polymers, and nanozymes is needed to improve sensitivity, stability, and reproducibility.

## 1 Introduction

Tuberculosis (TB) is a preventable and curable disease; however, it remains one of the greatest threats to global health. In 2023, TB likely reclaimed its position as the world’s leading cause of death from a single infectious agent, surpassing coronavirus disease (COVID-19) after three years and causing nearly twice as many deaths as HIV/AIDS. Each year, >10 million people fall ill with TB, and the number of new cases has been rising since 2021. Addressing this growing burden requires urgent and coordinated action [1].

Despite significant progress in the development of diagnostic platforms in recent years, early and accurate detection of TB remains difficult. Many patients are still diagnosed late or misdiagnosed, which contributes to ongoing transmission and poor clinical outcomes [2]. These shortcomings underscore the urgent need for innovative diagnostic strategies [3,4].

**Fig. 1:**
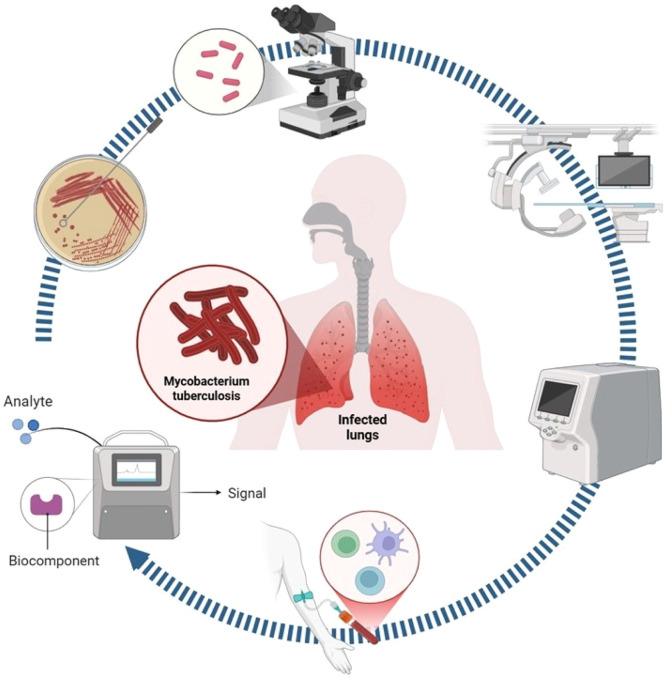
Overview of tuberculosis diagnostic approaches. This figure shows the trends in Mtb detection, illustrating the progression from conventional methods to nanotechnology-based approaches.

Fig. 1 presents the trends in Mycobacterium tuberculosis (Mtb) detection, illustrating the progression from conventional methods to nanotechnology-based approaches. The standard diagnosis of TB typically involves microscopic examination and culturing of Mycobacterium species from the sputum samples. However, these conventional methods are often time-consuming and may not be effective for early-stage detection of TB infection [4,5].

Although chest X-ray (CXR) is an important diagnostic tool for detecting TB in both symptomatic and asymptomatic individuals, it has several limitations [6]. CXR findings are often nonspecific, as many pulmonary diseases can produce radiographic patterns similar to those seen in TB, leading to false-positive or false-negative interpretations. The accuracy of CXR largely depends on the expertise of the examinator, which can result in significant variability between observers. In addition, CXR cannot reliably distinguish between active disease and TB infection, nor can it provide information about drug resistance. In resource-limited settings, access to high-quality imaging equipment and trained radiologists may also be restricted, further reducing its diagnostic utility. Therefore, while CXR serves as a valuable screening and supportive diagnostic tool, it should be complemented by microbiological or molecular tests to achieve definitive diagnosis. The Xpert MTB/RIF assay enables rapid molecular diagnosis and rifampicin resistance detection within two hours, but its limited sensitivity, occasional false positives, and reduced performance in polyresistant TB constrain its clinical utility [4,7]. Immunological tests such as the Tuberculin Skin Test (TST) and Interferon-Gamma Release Assays (IGRAs) are valuable tools for detecting tuberculosis infection. However, the TST is limited by cross-reactivity with Bacillus Calmette–Guérin (BCG) vaccination and non-tuberculous mycobacteria, as well as by delayed result interpretation. Although IGRAs offer higher specificity and faster turnaround times, they remain unable to differentiate between latent and active TB [[8], [9], [10]] and poorly predict TB progression [11].

**Fig. 2:**
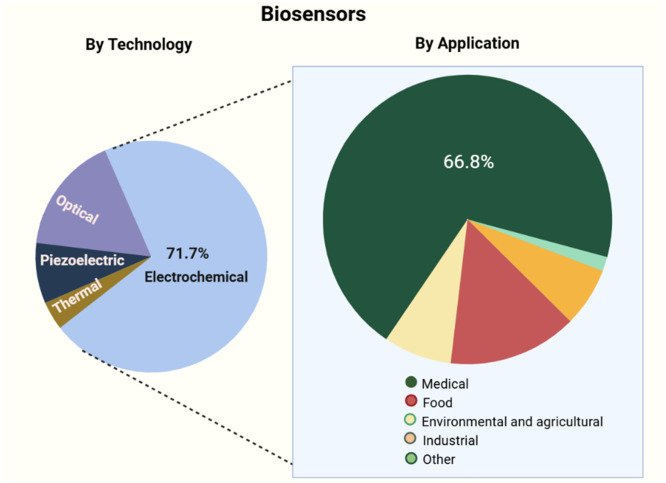
The global biosensors market, it shows that the electrochemical segment accounted for the largest share, capturing approximately 71.7% of the total market in 2024. Also the main application of electrochemical biosensors belong to medical section. https://www.grandviewresearch.com/industry-analysis/biosensors-market.

Recent diagnostic approaches increasingly employ biosensor-based assays, which continue to exhibit strong growth driven by rising demand for rapid and reliable diagnostic technologies [12]. Among the various technologies, the electrochemical segment accounted for the largest share, capturing approximately 71.7% of the total market in 2024 (Fig. 2). This segment’s dominance is attributed to its high sensitivity, cost-effectiveness, and suitability for point-of-care (POC) applications. Furthermore, it is expected to maintain its leading position with the fastest compound annual growth rate (CAGR) of 8.7% during the forecast period from 2025 to 2030. In terms of application, the medical segment accounted for the largest portion of market revenue, contributing about 66.8% in 2024. This substantial share is primarily driven by the widespread use of biosensors in clinical diagnostics, glucose monitoring, infectious disease detection, and other healthcare-related testing. The increasing prevalence of chronic diseases and the growing adoption of personalized medicine are further supporting market expansion within this segment.

Electrochemical immunosensors offer high specificity, simplicity, and miniaturization potential, requiring only small sample volumes and making them well-suited for on-site monitoring of MTB in POC settings. Their advantages of portability, reliability, and affordability make them promising tools for TB diagnostics, particularly in resource-limited environments [[13], [14], [15]]. In addition, electrochemical sensors provide rapid and straightforward responses, often within a minute. These fast detection capabilities make them especially valuable in emergencies, where obtaining timely results is critical.

Since the recognition event does not directly generate an electrochemical signal, two transduction principles are generally applied [16,17]. The most commonly used technique relies in the use of a redox-labelled secondary antibody that specifically recognizes the antigen–antibody complex rather than the individual antibodies. The presence of the redox probe generates a current at its characteristic redox potential, the intensity of which can be correlated with the number of antigens in the sample. Several techniques are available to measure the current and to quantify the antigen. For instance, cyclic voltammetry (CV) scans around the known redox potential of the probe visualizing the reduction and oxidation peak currents. It is an easy-to-apply method; however, in complex systems, background or parasitic currents may also be recorded. Therefore, one of the most commonly used technique is differential pulse voltammetry (DPV), in whichthe current is measured shortly before and after a pulsed potential change, thereby minimizing the influence of interfering background currents. The main disadvantage of this principle is the requirement for a labelled secondary antibody, which increases both cost and the number of handling steps. However, this dual-recognition strategy confirms the successful analyte capture and enables the achievement of low limits of detection.

To avoid the supplemental step, electrochemical impedance spectroscopy is a powerful and highly sensitive method allowing so-called “label-free” sensing. The principle is based on applying alternative currents at different frequencies around the redox potential of an electrochemical probe in solution. The recognition event, e.g., antigen capture, decreases the accessibility of the redox probe to the electrode surface, thereby increasing the charge transfer resistance at the electrode–solution interface. Although this approach offers high sensitivity, the absence of dual recognition increases the likelihood of non-specific binding from other compounds present in the sample. To reduce this effect, the electrode surface is typically passivated using suitable proteins or polymers.

**Fig. 3:**
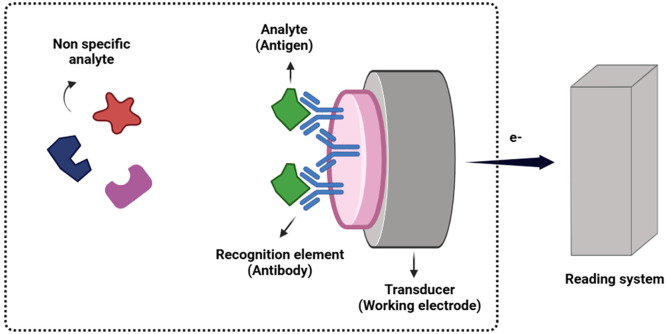
Schematic depiction of the basic design and components of an electrochemical immunosensor.

Regardless of the transduction technique, the receptor, commonly the antibody as shown in Fig. 3, has to be immobilized on the electrode surface [18]. However, this process can be challenging, as immobilization may affect biological activity and hinder the recognition event.

Various antibody immobilization strategies have been reported, including glutaraldehyde (Glu) cross-linking, EDC/NHS coupling, direct adsorption, and cysteamine hydrochloride (CH)–based attachment [[19], [20], [21], [22]].

Another controllable strategy is the immobilization via supramolecular interactions, where the receptor is produced with a specific guest molecule that couples with the host function on the electrode [23]. One of the most widely used system is the interaction between biotin (guest) and a avidin-family protein (guest). Each avidin has four “docking sites” for biotin and serves as an effective bridge for the immobilization of biotinylated receptors on biotinylated electrodes [24]. However, a drawback of this approach is the additional protein layer, which affect the electrochemical readout. More recent strategies have employed alternative molecular hosts like β-cyclodextrin, or metal coordination to immobilize biotinylated entities [25,26].

The challenge of obtaining effective and stable antibody immobilization is not specific to electrochemical immunosensors but is common to all immunosensor types. Among different types of immunosensors, electrochemical ones have the advantage of being quantitative and can be miniaturized to portable on-site analysis, while other types like optical POCs are generally less sensitive and not quantitative [27]. Optical quantification of the analyte with similar sensitivity still requires highly sophisticated instruments and qualified personnel. Additionally, electrochemical immunosensors are highly sensitive, capable of detecting analytes at femtomolar concentrations while maintaining a dynamic range extending from micromolar to femtomolar levels.

In this systematic review, we discuss electrochemical immunosensors and their potential role in improving TB diagnosis.

## 2 Methodology

This systematic review was conducted following the Preferred Reporting Items for Systematic Reviews and Meta-Analyses (PRISMA) guidelines. The review aimed to identify, evaluate, and summarize recent advances in the development and analytical performance of electrochemical immunosensors for TB detection. This review is registered in the International Prospective Register of Systematic Reviews database (PROSPERO registration number: CRD420251177591).

### 2.1 Search strategy

A comprehensive literature search was performed across PubMed, Scopus, and Web of Science databases to identify relevant studies. The search covered all articles published up to 7 September 2025. In addition to database searches, manual methods were applied to ensure completeness—specifically a backward citation search, in which the reference lists of included studies were screened to identify additional eligible publications. The search strategy combined keywords related to TB and electrochemical immunosensors, including ("tuberculosis"[MeSH Terms] OR "tuberculosis"[All Fields] OR ("*Mycobacterium tuberculosis*"[MeSH Terms] OR ("*Mycobacterium*"[All Fields] AND "tuberculosis"[All Fields]) OR "*Mycobacterium tuberculosis*"[All Fields]) OR "TB"[All Fields]) AND ((("electrochemical"[All Fields] OR "electrochemically"[All Fields]) AND ("biosensing techniques"[MeSH Terms] OR ("biosensing"[All Fields] AND "techniques"[All Fields]) OR "biosensing techniques"[All Fields] OR "biosensor"[All Fields] OR "biosensors"[All Fields] OR "biosensor s"[All Fields] OR "biosensoric"[All Fields] OR "biosensorics"[All Fields])) OR (("electrochemical"[All Fields] OR "electrochemically"[All Fields]) AND ("sensation"[MeSH Terms] OR "sensation"[All Fields] OR "sense"[All Fields] OR "senses"[All Fields] OR "sensed"[All Fields] OR "sensing"[All Fields] OR "sensings"[All Fields])) OR (("electrochemical"[All Fields] OR "electrochemically"[All Fields]) AND ("immunosensor"[All Fields] OR "immunosensors"[All Fields])) OR ("immunosensor"[All Fields] OR "immunosensors"[All Fields]) OR (("electrochemical"[All Fields] OR "electrochemically"[All Fields]) AND ("sensor"[All Fields]))).

All retrieved records were exported into EndNote reference management software for organization, screening, and removal of duplicate entries.

### 2.2 Selection process

Two independent reviewers screened all titles and abstracts based on predefined inclusion and exclusion criteria. Disagreements were resolved through discussion, and a third reviewer was consulted when consensus could not be reached. Full-text screening was then conducted to confirm eligibility. The final selection of studies was determined after thorough evaluation of the full-text articles.

### 2.3 Inclusion criteria

Studies were included if they met the following conditions:

1. Focused on TB, Mtb, or TB-related antigens.
2. Reported the use of electrochemical immunosensors for TB detection.
3. Evaluated the performance or analytical characteristics of electrochemical TB biosensors.

### 2.4 Exclusion criteria

Studies were excluded if they:

1. Focused on aptamer-based sensors or aptasensors, as previous systematic review has already extensively covered the use of aptamers in tuberculosis diagnosis through their interaction with specific antigens [28].
2. Targeted DNA-based detection (e.g., nucleic acid biosensors, target DNA, or peptide nucleic acid (PNA) biosensors).
3. Studied electrochemical biosensors for drug susceptibility testing, polymorphisms, or patient monitoring rather than direct TB antigen detection.
4. Targeted diseases other than TB, such as cancer, nontuberculous mycobacteria (NTM), or other pathogens.
5. Were not electrochemical immunosensor studies (e.g., optical, colorimetric, or purely molecular assays).

**Data extraction** and analysis.

Data were independently extracted by two reviewers from each included study. Data extraction involved. any discrepancies between reviewers were resolved through discussion. Authors were not contacted for missing or unclear data. Any discrepancies between reviewers were resolved through discussion.

The extracted data were systematically analyzed to provide a comprehensive review of the various electrochemical immunosensor approaches used for the detection of TB. The findings from the included studies were summarized based on the detection methods employed, and the components of the immunosensor such as electrode modifiers and labels. Summary measures and key performance indicators, including detection limit, and linear range, were reported where available to facilitate comparison across studies.

## 3 Results

**Fig. 4:**
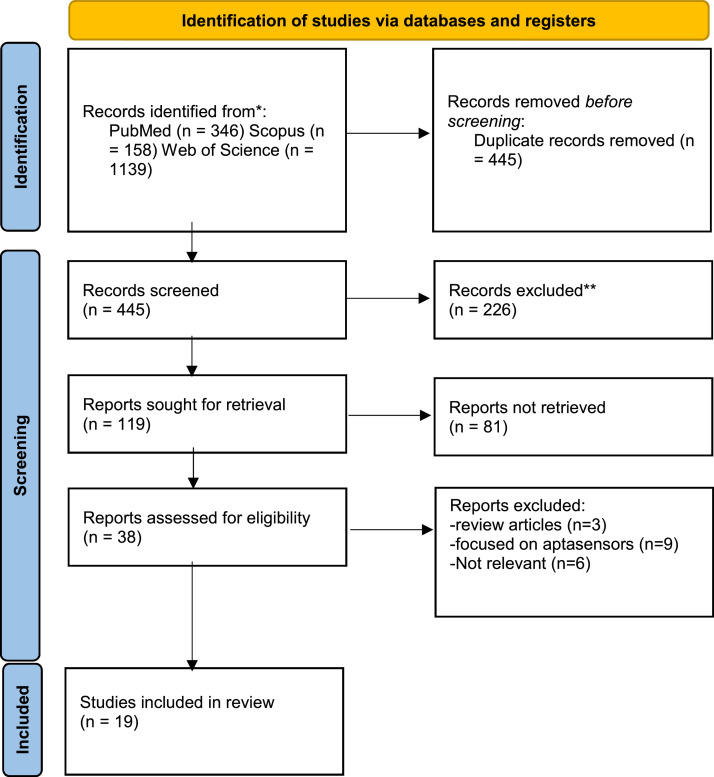
PRISMA flowchart detailing the systematic literature search and selection process.

A total of 1643 records were identified from PubMed (n = 346), Scopus (n = 158), Web of Science (n = 1139). After removing 445 duplicates, 445 records remained for screening. Of these, 226 were excluded. After full-text evaluation, 19 studies met the inclusion criteria and were included in the review (Fig. 4).

A total of 19 electrochemical immunosensor for detection of TB published between 2005 and 2025 were included in this review.

The characteristics of the included studies are summarized in Table 1. The study by Murphy et al. [29] was removed from Table 1, as it only demonstrates assay feasibility and does not provide sufficient analytical performance data for comparison.

**Table 1:** Summary of included studies using electrochemical immunosensors for TB detection.

Author	Year	Target analyte	Detection technique	Electrochemical label	Electrode modifier	Real sample matrix	Limit of detection (LOD)	Linear range	Stability	RSD Inter-assay (%)
María Díaz-González [30]	2005	Ag360	CV	AP	-	Not conducted	0.06 µg/mL	1–10 µg/mL	Stable for several weeks	< 8.1
		Ag360	SWV	AP	-	Not conducted	0.07 µg/mL	0.1–5 µg/mL		<8.7
		Ag231	CV/SWV	AP	-	Not conducted	0.001 µg/mL	0.005–0.1 µg/mL		<6.9 (CV), <9.1 (SWV)
Lisong Wang [47]	2012	anti-LAM	DPV	Au- SPA	-	Not conducted	5.3 ng/mL	15.6 to 1000 ng/mL	The response was 94.7% of the initial value after 10 days	<4.6
Subash C. B. Gopinath [45]	2016	16 kDa HSP	EIS	Label Free	APTES ZnO/AuNPs	Not conducted	1.6 ng/mL#	1.6 ng/mL– 16 µg/mL##	-	-
Mohamed Fethi Diouani [31]	2017	ESAT6	SWV	Label Free	-	Not conducted	7.1 ng/mL	10–1000 ng/mL	-	<1.93
Panuvat Chutichetpong [43]	2018	MPT64	Amperometry	HRP	-	Sputum	0.43 ng mL	0.3–50 ng/mL and 50–1000 ng/mL	The signal was lost after 5 days of storage at 4 °C	-
Dan Gou [42]	2018	MPT64	CV	GO@Fe3O4@Pt	-	Serum	0.34 fg/mL	5.0 fg/mL −1.0 ng/mL	The response was 97.31%, 95.21%, and 90.23% of the initial value after 5, 10, and 15 days of storage at 4 °C	-
Umi Zulaikha Mohd Azmi [38]	2018	CFP10	CV	Fe3O4@Au	GP/PANI	Sputum	15 ng/mL	20–100 ng/mL	-	0.55
Lemma Teshome Tufa [36]	2018	CFP10	DPV	Au	Fe3O4–Ag/GQD NPs	Urine	0.33 ng/ml	5–500 ng/ml	-	4.2
Noremylia Mohd Bakhori [40]	2019	CFP10-ESAT6	DPV	Catalase	CdSe/ZnSQD/Si NPs	Not conducted	0.15 ng/mL	40 to 100 ng/mL	-	1.45
Umi Zulaikha Mohd Azmi [39]	2021	CFP10-ESAT6	DPV	Fe3O4@Au	GP/PANI	Sputum	1.5 ng/mL	10- 500 ng/mL	The response was >90% of the initial value at 4 °C for 28 days	3.4
Rishabh Anand Omar [32]	2021	ESAT6	CV	Label free	rGO-Ni NPs and PANI film	Plasma	1.0 ng/mL	1–100 ng/mL	Stable for > 6 months at 0 °C	<2
Syazana Ameera Syed Amri [33]	2021	ESAT6-Like Protein EsxB2	DPV	Fe3O4@Au	rGO-APTES	Not conducted	1.32 ng/mL	0–9.0 µg/mL	-	6.4
Yanping Xia [34]	2023	ESAT6	EIS	Label free	N-GO	Serum	7.0 pg/mL	0.01–100 ng/mL	The response was >93% of the initial response after 2 months at 4 °C	4.5
Thimpika Pornprom [46]	2024	Hsp 16.3	SWV	Label free	Graphene-AuNPs/ GCOOH	Serum	0.01 ng/mL	0.01–30 ng/mL	The response was >90% of the initial value after 3 weeks at 4 °C, 76% after 5 weeks	<3.4
Luisa Vogado Ribeiro [41]	2024	CFP10-ESAT6	EIS	Label free	APTES	Not conducted	4.80 ng/mL	0.5 ng/mL to 50 ng/mL	-	24
Dinesh R Rotake [44]	2025	LAM	CV/ SWV	Label free	MWCNT–ZnO-NFs	Urine	77 fg/ml	1 pg/ml–6 ng/ml	The response was >88% of the initial value after 42 days	6.19^⸹^
Xiu-An Ye [35]	2025	ESAT6	DPV	Label free	PEDOT-COOH	Plasma	47.08 pg/mL	0.8–1.7 × 103 ng/mL	The response was 90.1% of its initial value after 20 days	1.21
Seifert [37]	2024	CFP10	Amperometry	HRP	-	Serum	44 pg/mL	11–59 ng/mL*	No systematic decrease over 30 days	<5.9
						Urine	77 pg/mL	36–88 ng/mL**		

#100 fM.

##100 fM–1 nM.

*1.1 to 5.9 nM.

**3.6–8.8 nM.

⸹ Value derived from the article’s reported data 0.57×100/9.21=6.19.

CV: Cyclic voltammetry.

DPV: Differential pulse voltammetry.

SWV: Square-wave voltammetry.

EIS: Electrochemical impedance spectroscopy.

LAM: lipoarabinomannan.

HSP: Heat shock protein.

SPA: Staphylococcal protein A.

AP: Alkaline phosphatase.

HRP: Horseradish Peroxidase.

NPs: Nanoparticles.

GQD: Graphene quantum dot.

QD: Quantum dot.

GP/PANI: Graphene/polyaniline.

Si NPs: Silica nanoparticles.

Ni NPs: Nickel nanoparticles.

PANI: Polyaniline.

PEDOT: Poly 3,4- ethylenedioxythiophene.

rGO: Reduced graphene oxide.

N-GO: N-doped graphene oxide.

GCOOH: Carboxyl graphene.

APTES: 3-aminopropyltrimethoxysilane.

MWCNT–ZnO—NFs: Multiwall-carbon nanotube-zinc oxide nanofibers.

PEDOT-COOH: Poly(3,4-ethylene dioxythiophene) with carboxyl groups.

PG: Protein G.

### 3.1 Mtb targets in TB electrochemical immunosensors

The studies targeted a range of Mtb antigens, including Ag85 [29,30], ESAT6 [[31], [32], [33], [34], [35]], CFP10 [[36], [37], [38]], CFP10-ESAT6 proteins [[39], [40], [41]], MPT64 [42,43], lipoarabinomannan (LAM) [44], and heat shock proteins (Hsp16) [45,46]. Additionally, one study focused on the detection of anti-LAM [47].

### 3.2 Electrode modification strategies in TB electrochemical immunosensors

A range of electrode modifiers has been employed to enhance the electrochemical performance, selectivity, and sensitivity of biosensors. The modifiers can be broadly classified into metallic nanoparticles (NPs) and their nanocomposite with carbon-based nanomaterials, conductive polymers and their nanocomposite with carbon-based nanomaterials**,** and ternary nanocomposites that combine all of these materials.

Several studies have demonstrated the effects of metallic NPs or their combination with carbon-based materials in enhancing the sensitivity, stability, and electron-transfer efficiency of biosensors. Metallic NPs such as Au, Ag, Ni, and metal oxides, are widely used due to their outstanding conductivity, high surface-area-to-volume ratio, and catalytic properties. For instance, Murphy et al. [29] employed gold nanoparticles (Au NPs) to improve electron transfer efficiency, leading to improved analytical sensitivity. Gopinath et al. [45] synthesized a ZnO/Au NP composite, in which ZnO provided biocompatibility and enhanced adsorption sites, while Au NPs facilitated efficient charge transfer. Similarly, Mohd Bakhori et al. [40] incorporated Cadmium Selenide Zinc Sulfide Quantum Dots Silicon nanoparticles (CdSe/ZnSQD/Si NPs), leveraging their high electron mobility to boost electrochemical responses. In general, combining metallic NPs with carbon-based materials often produces synergistic effects. Pornprom et al. [46] developed a graphene–Au NP hybrid for enhanced conductivity and surface area. Rotake et al. [44] reported a multi-walled carbon nanotube (MWCNT)/ZnO hybrid system that benefits from the fast electron-transport pathways of MWCNTs and the high surface activity of ZnO. Beyond metallic nanomaterials, conductive polymers and graphene derivatives have also played an important role in creating efficient transducers. Conductive materials are valued for their processability, stability, and ability to form uniform coatings that promote electron mobility. Ye et al. [35] utilized poly(3,4-ethylenedioxythiophene) (PEDOT) as a conductive layer to enhance signal transduction. Xia et al. [34] applied nitrogen-doped graphene oxide (N-GO), where nitrogen doping introduces additional active sites and increases electron density, improving the electron-transport rate. Amri et al. [33] reported that electrodes modified with rGO offer enhanced conductivity and improved biorecognition due to their large electroactive surface. In addition, to exploit the synergistic effects between conductive polymers and graphene, Mohd Azmi and colleagues [38,39] developed graphene/PANI composites in two separate studies.

More advanced ternary architectures have also been explored. Omar et al. [32] employed a reduced graphene oxide (rGO)–Ni NP–polyaniline (PANI) film. Tufa et al. [36] also fabricated a ternary nanocomposite comprising graphene quantum dots (GQDs), Fe₃O₄, and Ag nanoparticles, achieving enhanced electrochemical activity.

### 3.3 Detection strategies in TB electrochemical immunosensors

Detection strategies in TB electrochemical immunosensors are generally categorized as label-based or label-free. A growing number of studies have focused on label-free biosensors [29,31,32,34,35,41,[44], [45], [46]], motivated by its operational simplicity, reduced preparation steps, lower cost, and avoidance of potential steric hindrance caused by bulky labels. Label-free strategies often rely heavily on electrode surface modification, where conductive nanomaterials and biocompatible interfaces amplify changes in impedance or current caused by antigen–antibody binding.

**Fig. 5:**
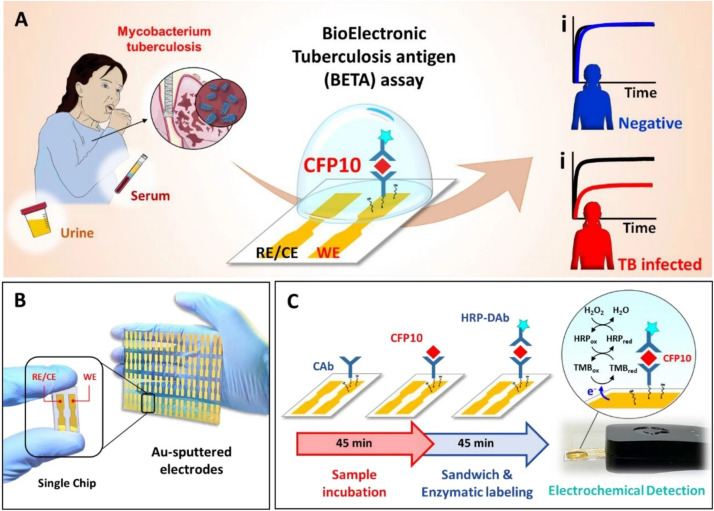
Schematic of the bioelectronic TB antigen (BETA) assay workflow for CFP10 detection. (A) Image of the electrochemical immunosensor (B) two gold-sputtered electrodes—one serving as the working electrode (WE) and the other as the combined reference/counter electrode (RE/CE). (C) the two-step sandwich-type immunoassay involving sequential 45-minute incubations of the unprocessed clinical sample and the HRP-tagged detector antibody. The electrochemical reaction of the H₂O₂/HRP/TMB redox system generates a measurable current proportional to antigen concentration. Reproduced from Seifert et al. study [37], under the terms of the Creative Commons Attribution 4.0 International License.

In contrast, label-based biosensors incorporated enzymatic or nanomaterial-based labels to amplify signals. Some studies used enzymes such as horseradish peroxidase (HRP) (Fig. 5) [37,43], alkaline phosphatase [30] or catalase [40], each capable of catalyzing reactions that generate measurable electroactive species. Gou et al. employed a GO@Fe₃O₄@Pt nanocomposite as a nanozyme mimic of peroxidase activity [42], providing catalytic amplification without the stability limitations of natural enzymes. Au-based nanoparticles were also used as labels in several studies [33,36,38,39,47].

### 3.4 Detection limits (LOD) of TB electrochemical immunosensors

Improvements in both transducer materials and detection strategies have led to substantial advancements in the detection limits of TB immunosensors. In 2005, Díaz-González et al. [30], employed traditional alkaline phosphatase (AP)-based labeling systems, achieving detection limits in the microgram range (0.001–0.07 µg/mL). Although effective at that time, these LODs were limited by less sophisticated surface engineering and basic enzyme-label amplification. Subsequent studies demonstrated that electrode surface modification can significantly improve sensor performance in both label-free [29,32,34,35,41,[44], [45], [46]] and label-based detection methods [33,36,[38], [39], [40]]. Nevertheless, some reports achieved signal amplification solely through the use of enzymatic or non-enzymatic labels, without incorporating any electrode surface modification.

### 3.5 Stability and reproducibility of the immunosensor

Some studies have investigated the reproducibility [[30], [31], [32], [33], [34], [35], [36], [37], [38], [39], [40], [41],^44^,^46^,^47^] and stability [30,32,34,35,37,39,[42], [43], [44],^46^,^47^] of immunosensors. Reproducibility is typically evaluated by measuring the same antigen—at one or multiple concentrations—using sensors fabricated under identical conditions, with the relative standard deviation (RSD) used to assess measurement consistency (Table 1). Stability is generally determined by monitoring signal retention over a defined period, such as days or weeks.

## 4 Discussion

Recent advancements in electrochemical immunosensors for TB detection have demonstrated remarkable improvements in sensitivity, detection range, and biocompatibility through the use of nanomaterials, as electrochemical labels and/or advanced electrode modifiers.

Nanomaterials have become attractive in bioassay systems, significantly improving sensitivity and detection limits. Their unique electrical and catalytic properties offer effective alternatives to conventional transduction methods. Moreover, combining different nanomaterials—such as metal NPs, carbon-based nanostructures, and conductive polymers—can produce synergistic effects that further enhance biosensor performance, leading to larger surface area, higher stability, faster electron transfer, and greater analytical sensitivity [48,49].

Most studies conducted up to 2018 were based on bare screen-printed electrodes (SPEs), typically made of either gold [31,37] or carbon [30,43,47]. As expected, these biosensors did not achieve detection limits as low as those obtained with nanomaterial-modified electrodes.

Overall, the modifiers can be divided into three main categories. The first category includes studies that used only metallic NPs [29,40,45] or a combination of metallic NPs with carbon-based nanomaterials such as graphene derivatives [46] and MWCNTs [44]. The second category consists of modifiers based on conductive polymers [35], graphene derivatives [33,34], or their combinations [38,39]. The third category encompasses hybrid materials that integrate all three components—polymers, metal NPs, and graphene derivatives [32,36,46].

As mentioned, some studies have shown that NPs and their hybrids with carbon-based materials significantly enhance biosensor sensitivity, stability, and electron-transfer efficiency.

However, metallic NPs alone face notable limitations. They tend to aggregate, which reduces the effective surface area and diminishes reproducibility. Some metal oxides have relatively low electrical conductivity, limiting electron transfer. Additionally, long-term stability can be poor due to oxidation or dissolution, and their surface chemistry often requires further modification to immobilize biomolecules effectively [50]. To address these drawbacks, metallic NPs are frequently combined with carbon-based materials such as graphene or carbon nanotubes, which provide conductive networks, prevent aggregation, and enhance electron transfer. For instance, Rotake et al. [44] reported that MWCNT/ZnO hybrids leveraged the high electron transport of MWCNTs and the catalytic activity of ZnO, achieving a LOD of 77 fg/mL compared to 1.6 ng/mL for ZnO/Au NPs [45], demonstrating the significant enhancement from the synergistic combination. Similarly, combining Au NPs with graphene further reduced the LOD to 0.01 ng/mL [46], emphasizing the synergistic advantages of these hybrid systems.

In addition, conductive polymers and graphene-based materials play a key role in the design of efficient transducer due to their stability, processability, and ability to form uniform conductive films. Materials such as PEDOT, nitrogen-doped graphene oxide, and rGO enhance electron transport and provide large electroactive surfaces for improved biorecognition. Studies have demonstrated that these materials, individually, can achieve respectable detection limits. For example, N-GO achieves an LOD of 7 pg/mL[34], and PEDOT-COOH achieves ∼47 pg/mL [35].

Despite their advantages, polymers and carbon materials also have limitations. Polymers may suffer from limited mechanical stability, swelling in aqueous media, and relatively slow electron-transfer kinetics compared to metallic NPs. Carbon materials alone may show site-to-site variability, require doping or functionalization to introduce sufficient active sites, and can be prone to aggregation in suspension or on surfaces. To overcome these drawbacks, combining conductive polymers with carbon materials has been shown to improve performance [51]. Mohd Azmi et al. [38,39] demonstrated that graphene/PANI composites achieved LODs in the range of 1.5–15 ng/ mL, while also providing enhanced reproducibility (RSD as low as 0.55%). These improvements are attributed to uniform polymer distribution on the graphene scaffold, π–π interactions that stabilize the polymer network, flexible charge-transfer pathways, and homogeneous accessibility of active sites.

The integration of carbon materials, metallic NPs, and conductive polymers into ternary hybrid composites can produce synergistic effects that surpass the performance of individual components or binary composites. In these systems, carbon scaffolds provide a high-surface-area conductive network, metallic NPs supply abundant catalytic sites and facilitate electron transfer, and conductive polymers create a flexible, stable matrix for charge transport and biomolecule immobilization.

Notably, a label-based graphene/PANI system with Fe3O4@Au nanoparticles achieved an LOD of 15 ng/mL [38]. In contrast, a label-free system combining PANI, Ni NPs, and rGO further reduced the LOD to 1 ng/mL [32], with both studies employing CV as the detection technique. These findings highlight that ternary composites can deliver high sensitivity even in the absence of labeling strategies.

Beyond sensitivity, such ternary composite exhibit remarkable stability and reproducibility. Sensors maintain performance for over six months, with RSD values below 2%. This is attributed to strong interfacial interactions (π–π stacking and metal–polymer coordination), metallic NPs acting as anchoring points for polymers and biomolecules, uniform distribution of active sites within a three-dimensional network, and enhanced overall conductivity that reduces background noise and increases the signal-to-noise ratio.

Although metallic NPs, carbon-based materials, and conductive polymers each offer distinct advantages, they also possess inherent limitations that may hinder sensitivity, stability, or reproducibility. The formation of hybrid systems—whether binary or ternary—enables synergistic interactions among components, effectively addressing these challenges and enhancing overall performance.

In the label-free format, the best performance was obtained from the sensor employing MWCNT–ZnO nanofibers as the modifier [44], achieving a LOD of 77 fg/mL. However, comparing the effect of modifiers in label-based methods is challenging, as the presence of a label can independently influence the overall sensitivity.

Overall, the trend demonstrates a clear shift from enzyme-labeled to label-free immunosensors, which are simpler to fabricate and more suitable for POC applications. To maintain high sensitivity in these systems, nanostructured modifiers have been introduced not only to increase the effective surface area but also to enhance surface conductivity and facilitate electron transfer.

All label-free electrochemical sensing strategies discussed in this review operate based on an inhibition-based (signal-off) mechanism. In these systems, the binding of the target antigen onto the electrode surface forms a physical and/or electrostatic barrier that hinders the diffusion of redox probes and/or impedes interfacial electron transfer. As a result, a decrease in faradaic current is typically observed in voltammetric techniques [29,31,32,35,44,46], while an increase in charge transfer resistance (Rct) is detected in electrochemical impedance spectroscopy (EIS) [34,41,45]. Despite the apparent difference in signal direction, both responses originate from the same underlying phenomenon, namely the suppression of electrochemical activity at the electrode interface.

While the use of AP as a labeling enzyme dates back to the first study in 2005, subsequent research (2012–2019) increasingly employed more common and commercially available peroxidase enzymes such as HRP and catalase. However, the performance of enzyme-based systems is often constrained by the loss of enzymatic activity during immobilization [52]. By eliminating the need for enzymatic labels, this strategy reduces assay complexity, cost, and potential instability associated with enzyme-based systems [47].

As an interesting alternative, Gou and his colleagues [42] utilize peroxidase-mimicking nanozyme (GO@Fe3O4@Pt), which not only improved the LOD, but also enhanced stability due to the use of non-enzymatic catalyst. This approach demonstrated outstanding analytical performance, achieving an LOD of 0.34 fg/mL and a linear range from 5.0 fg/mL to 1.0 ng/mL. The best analytical performance has been reported for this study with LOD of 0.34 fg/ml and linear range between 5.0 fg/mL to 1.0 ng/mL. In addition, there are some reports that used nanomaterials for amplifying signal in an non-enzymatic manner; however, most of these relied primarily on Au as label.

Secretory proteins of Mtb have attracted increasing attention as potential biomarkers for the early diagnosis of TB [15]. Among the various target antigens, ESAT6, CFP10, and CFP10–ESAT6 fusion proteins were the most commonly used due to their strong immunogenicity and diagnostic relevance [[31], [32], [33], [34], [35], [36], [37],[39], [40], [41]]. Studies using MPT64 [42,43] and LAM [44] also demonstrated high analytical performance, particularly in clinically relevant matrices such as serum, plasma, sputum, and urine. Approximately one-third of TB patients are seronegative for any single antigen [53], which greatly restricts the diagnostic utility of antibody-based tests when used alone. To address these shortcomings, employing a combination of different antigens may provide a more comprehensive and reliable approach.

**Limitations and applications of electrochemical biosensors** Despite notable progress in electrochemical immunosensor development for TB detection, current research still exhibits several conceptual and methodological limitations. A considerable number of studies continue to rely on bare or unmodified electrodes, where antibodies are immobilized through simple drop-casting procedures. Such an approach often results in poor control over antibody orientation, weak binding stability, and inefficient electron transfer, ultimately compromising analytical performance.

Although carbon-based nanomaterials offer excellent conductivity and biocompatibility, their use in TB electrochemical immunosensors is still limited. Graphene and its derivatives, including GO and rGO, have dominated most reported works due to their high surface area and ease of functionalization. However, apart from a single study employing MWCNTs, the diversity of carbon-based nanostructures remains largely unexplored. This narrow focus overlooks the potential of emerging carbonaceous materials such as MXenes, carbon nanohorns, carbon nanofibers, and doped graphene frameworks, all of which possess superior electrical conductivity, abundant active sites, and flexible surface chemistry for biomolecule conjugation.

Similarly, the variety of conducting polymers used as immobilization matrices remains restricted mainly to PANI and PEDOT. These polymers undoubtedly provide desirable conductivity and mechanical stability, but other electroactive and functionalized polymers could offer enhanced functional group density, improved biorecognition efficiency, and higher sensor reproducibility.

Another critical gap concerns the use of nanozymes as signal amplification elements. Although nanozymes have rapidly evolved as robust, tunable, and cost-effective enzyme mimics, only one report has described a nanozyme-based TB immunosensor. Given the rapid advancement of MOF-based nanozymes, metal oxide nanozymes, and hybrid nanocatalysts with tailored catalytic activity, this area remains significantly underexplored. The integration of such materials could provide enzyme-free systems with superior catalytic durability and enhanced signal transduction efficiency.

In addition, in most nanomaterial-based electrochemical labels, the signal generation still relies on the redox reaction of gold, typically through the redox labelled AuNPs. While Au provides excellent conductivity and biocompatibility, it is costly and susceptible to potential shifts or instability under repeated electrochemical cycling. More importantly, the overreliance on the Au redox process limits the design flexibility of signal transduction mechanisms. Alternative redox-active probes, such as ferrocene derivatives, Prussian blue analogs, transition-metal complexes (e.g., Ru-, Cu-, or Co-based species), and stable organic redox mediators, could serve as cheaper, more stable, and tunable electrochemical reporters, enabling multi-analyte detection and improved long-term sensor performance.

Ongoing advances in detection technologies, such as POC diagnostic devices and digital assay platforms, provide promising opportunities to further enhance their applicability. With continued optimization, these integrated diagnostic strategies could serve as valuable complements to existing gold-standard methods or potentially evolve into stand-alone tools for TB diagnosis, particularly in resource-limited settings where rapid and affordable testing is critically needed.

On the other hand, the lack of comprehensive interference assessment may reduce the reliability, accuracy, and comparability of the reported sensor performances. Accordingly, future studies should incorporate detailed evaluations of matrix effects and develop robust strategies to effectively minimize interference in real sample analysis.Developing reliable non-sputum-based rapid tests for TB, however, remains a major challenge. This difficulty stems from the disease’s primary localization within the lung parenchyma and its complex pathobiology, which limit the availability of detectable biomarkers in peripheral samples [54]. The limited availability of reliable biomarkers further complicates this effort, as only a small fraction of potential biomarkers identified in early research successfully progress to clinically applicable diagnostic tests. Despite these challenges, several promising non-sputum approaches using urine, tongue swabs, breath, blood, or stool are emerging [54]. Notably, the AlereLAM urine test for people living with HIV and the Xpert MTB/RIF stool test for children have already been endorsed by the WHO [55]. Continued evaluation and optimization of such assays will be crucial to expanding diagnostic access, especially for early detection of TB.

## 5 Conclusion

Electrochemical immunosensors offer a rapid, sensitive, and specific approach for detecting TB. Advances in nanomaterials and conductive polymers have greatly improved sensor performance. However, most studies still rely on conventional materials and basic immobilization strategies, overlooking the potential of new-generation nanomaterials and redox systems. Future research should focus on integrating advanced carbon-based modifiers, diverse functional polymers, and emerging nanozymes to enhance sensitivity, stability, and reproducibility. Furthermore, replacing redox-labelled AuNPs with more stable and cost-effective redox probes could pave the way toward next-generation TB immunosensors with improved analytical and practical performance. In summary, continued innovation in materials and device design will be crucial to translate these technologies into reliable POC tools for early TB diagnosis.

## Funding

This work was supported by the National Science Centre, Poland, under the OPUS program (UMO-2024/53/B/NZ7/03922 Grant No. 2024/53/B/NZ7/03922) and 32/007/SDU/10-07-01 implemented under the Research University program of the Silesian University of Technology, Gliwice, Poland. For Open Access, the author has applied a CC-BY public copyright license to any Author Accepted Manuscript (AAM) version arising from this submission.

## CRediT authorship contribution statement

**Sasan Radfar:** Writing – review & editing, Writing – original draft, Visualization, Validation, Methodology, Investigation, Conceptualization. **Mostafa Teymuri:** Writing – review & editing, Validation, Methodology, Investigation, Formal analysis. **Michael Holzinger:** Writing – review & editing, Validation, Supervision, Methodology. **Shima Mahmoudi:** Writing – review & editing, Writing – original draft, Visualization, Validation, Supervision, Software, Project administration, Methodology, Investigation, Funding acquisition, Formal analysis, Conceptualization.

## Declaration of competing interest

The authors declare that they have no known competing financial interests or personal relationships that could have appeared to influence the work reported in this paper.

## Data Availability

Data will be made available on request.

## References

[1] Bagcchi S. (2023). WHO's global tuberculosis report 2022. Lancet Microbe..

[2] Dong B., He Z., Li Y., Xu X., Wang C., Zeng J. (2022). Improved conventional and new approaches in the diagnosis of tuberculosis. Front. Microbiol..

[3] Yang X.R., Fan S.H., Ma Y.H., Chen H., Xu J.F., Pi J. (2022). Current progress of functional nanobiosensors for potential tuberculosis diagnosis: the novel way for TB control?. Front. Bioeng. Biotechnol..

[4] Mahmoudi S., García M.J., Drain P.K. (2024). Current approaches for diagnosis of subclinical pulmonary tuberculosis, clinical implications and future perspectives: a scoping review. Expert. Rev. Clin. Immunol..

[5] Mahmoudi S., Hamidi M., Drain P.K. (2024). Present outlooks on the prevalence of minimal and subclinical tuberculosis and current diagnostic tests: a systematic review and meta-analysis. J. Infect. Public Health.

[6] Kontsevaya I., Cabibbe A.M., Cirillo D.M., DiNardo A.R., Frahm N., Gillespie S.H. (2024). Update on the diagnosis of tuberculosis. Clin. Microbiol. Infect..

[7] Mahmoudi S., Sadegh Moghaddasi A.H. Evaluation of truenat assays for the diagnosis of pulmonary and extrapulmonary tuberculosis: a systematic review and meta-analysis. 10.1080/14787210.2024.2389876 39115877

[8] Mahmoudi S., Hosseini Sharif S.M.S., Mahmoudi S., Nourazar S. Diagnostic accuracy of QuantiFERON-TB gold plus with chemiluminescence immunoassay: a systematic review and meta-analysis evaluating the diagnostic accuracy of QIAreach QuantiFERON-TB compared to QuantiFERON-TB gold plus for tuberculosis: a systematic review and meta-analysis.

[9] Mahmoudi S., Nourazar S. Evaluating the diagnostic accuracy of QIAreach QuantiFERON-TB compared to QuantiFERON-TB gold plus for tuberculosis: a systematic review and meta-analysis. 10.1038/s41598-024-65663-4 PMC11196697 38914731

[10] Mamishi S., Pourakbari B., Hosseinpour Sadeghi R., Marjani M., Mahmoudi S.A.-.O. Diagnostic accuracy of the IFN-γ release assay using RD1 immunodominant T-cell antigens for diagnosis of extrapulmonary tuberculosis. LID - fnae023 [pii] LID - 10.1093/femsle/fnae023. 10.1093/femsle/fnae023 38533666

[11] Gupta R.K., Lipman M., Jackson C., Sitch A.J., Southern J., Drobniewski F. (2020). Quantitative IFN-γ release assay and tuberculin skin test results to predict incident tuberculosis. a prospective cohort study. Am. J. Respir. Crit. Care Med..

[12] Pornprom T., Pakamwong B., Sangswan J., Punkvang A., Thongdee P., Suttisintong K. (2026). Contactless point-of-care detection of latent tuberculosis biomarker Hsp16. 3 using a high-sensitivity magnetoimpedance biosensor. Sens. Actuators, A.

[13] Shanbhag M.M., Manasa G., Mascarenhas R.J., Mondal K., Shetti N.P. (2023). Fundamentals of bio-electrochemical sensing. Chem. Eng. J. Adv..

[14] Golichenari B., Nosrati R., Farokhi-Fard A., Maleki M.F., Hayat S.M.G., Ghazvini K. (2019). Electrochemical-based biosensors for detection of mycobacterium tuberculosis and tuberculosis biomarkers. Crit. Rev. Biotechnol..

[15] Joshi H., Kandari D., Maitra S.S., Bhatnagar R. (2022). Biosensors for the detection of mycobacterium tuberculosis: a comprehensive overview. Crit. Rev. Microbiol..

[16] Ochoa-Ruiz A.G., Parra G., López-Espinoza D., Astudillo P., Galyamin D., Sabaté N. (2023). Electrochemical immunosensors: the evolution from ELISA to EμPADs. Electroanalysis.

[17] Kumar S., Kalkal A. (2021). Nanotechnology in Cancer Management.

[18] Asal M., Özen Ö., Şahinler M., Baysal H.T., Polatoğlu İ. (2019). An overview of biomolecules, immobilization methods and support materials of biosensors. Sens. Rev..

[19] Halámek J., Hepel M., Skládal P. (2001). Investigation of highly sensitive piezoelectric immunosensors for 2, 4-dichlorophenoxyacetic acid. Biosens. Bioelectron..

[20] Puertas S., de G., Villa M., Mendoza E., Jiménez-Jorquera C., de la Fuente J.M., Fernández-Sánchez C. (2013). Improving immunosensor performance through oriented immobilization of antibodies on carbon nanotube composite surfaces. Biosens. Bioelectron..

[21] Zhu Q., Du J., Feng S., Li J., Yang R., Qu L. (2022). Highly selective and sensitive detection of glutathione over cysteine and homocysteine with a turn-on fluorescent biosensor based on cysteamine-stabilized CdTe quantum dots. Spectrochim. Acta, Part A.

[22] Huang J., Xie Z., Xie L., Luo S., Zeng T., Zhang Y. (2022). Explore how immobilization strategies affected immunosensor performance by comparing four methods for antibody immobilization on electrode surfaces. Sci. Rep..

[23] Holzinger M., Le Goff A., Cosnier S. (2014). Supramolecular immobilization of bio-entities for bioelectrochemical applications. New J. Chem..

[24] Wilchek M., Bayer E.A. (1988). The avidin-biotin complex in bioanalytical applications. Anal. Biochem..

[25] Holzinger M., Singh M., Cosnier S. (2012). Biotin− β-cyclodextrin: a new host–Guest system for the immobilization of biomolecules. Langmuir.

[26] Baur J., Holzinger M., Gondran C., Cosnier S. (2010). Immobilization of biotinylated biomolecules onto electropolymerized poly (pyrrole-nitrilotriacetic acid)–Cu2+ film. Electrochem. Commun..

[27] Pour S.R.S., Calabria D., Emamiamin A., Lazzarini E., Pace A., Guardigli M. (2023). Electrochemical vs. optical biosensors for point-of-care applications: a critical review. Chemosensors.

[28] Isaei E., Sobhanipoor M.H., Rahimlou M., Firouzeh N. (2024). The application of aptamer in tuberculosis diagnosis: a systematic review. Trop. Dis. Travel. Med. Vaccines..

[29] Murphy B., Dempsey E. (2020). Evaluation of an Ag85B immunosensor with potential for ElectrochemicalMycobacterium TuberculosisDiagnostics. ECS J. Solid State Sci. Technol..

[30] Díaz-González M., González-García M.B., Costa-García A. (2005). Immunosensor for mycobacterium tuberculosis on screen-printed carbon electrodes. Biosens. Bioelectron..

[31] Diouani M.F., Ouerghi O., Refai A., Belgacem K., Tlili C., Laouini D. (2017). Detection of ESAT-6 by a label free miniature immuno-electrochemical biosensor as a diagnostic tool for tuberculosis. Mater. Sci. Eng. C-Mater. Biol. App..

[32] Omar R.A., Verma N., Arora P.K. (2021). Development of ESAT-6 based immunosensor for the detection of mycobacterium tuberculosis. Front. Immunol..

[33] Amri S.A.S., Yusof N.A., Abdullah J., Abd Rahman S.F., Azmi U.Z.M (2021). Enhancement of electrochemical properties using iron oxide-gold nanocomposite for tuberculosis detection based on rGO-APTES modified screen-printed electrode. IEEE Sens. J..

[34] Xia Y.P., Zhu H.B., Liu R.X., Li X., Xie X.L., Zhao J. (2023). Picogram level electrochemical impedimetric immunosensor for monitoring mycobacterium tuberculosis based on specific and sensitive ESAT-6 monoclonal antibody. Talanta.

[35] Ye X.A., Lin S.H., Chen L.Y., Li J.R., Chiu H.W., Pan S.W. (2025). Innovative polymer-based electrochemical platform for detecting ESAT-6 in human blood for pulmonary tuberculosis diagnosis. Sens. Biosensing. Res..

[36] Tufa L.T., Oh S., Tran V.T., Kim J., Jeong K.J., Park T.J. (2018). Electrochemical immunosensor using nanotriplex of graphene quantum dots, Fe3O4, and Ag nanoparticles for tuberculosis. Electrochim. Acta.

[37] Seifert M., Vargas E., Montiel V.R.V., Wang J., Rodwell T.C., Catanzaro A. (2021). Detection and quantification of mycobacterium tuberculosis antigen CFP10 in serum and urine for the rapid diagnosis of active tuberculosis disease. Sci. Rep..

[38] Mohd Azmi U.Z., Yusof N.A., Kusnin N., Abdullah J., Suraiya S., Ong P.S. (2018). Sandwich electrochemical immunosensor for early detection of tuberculosis based on graphene/polyaniline-modified screen-printed gold electrode. Sensors. (Basel).

[39] Mohd Azmi U.Z., Yusof N.A., Abdullah J., Alang Ahmad S.A., Fatin F.N., Ahmad Raston N.H. (2021). Portable electrochemical immunosensor for detection of mycobacterium tuberculosis secreted protein CFP10-ESAT6 in clinical sputum samples. Microchim. Acta.

[40] Mohd Bakhori N., Yusof N.A., Abdullah J., Wasoh H., Ab Rahman S.K., Abd Rahman S.F (2019). Surface enhanced CdSe/ZnS QD/SiNP electrochemical immunosensor for the detection of mycobacterium tuberculosis by combination of CFP10-ESAT6 for better diagnostic specificity. Mater. (Basel).

[41] Ribeiro L.V., Cancino-Bernardi J., CdA R., Machado T.R., Tuesta M.A., Zucolotto V. (2024). Exploring electrochemical impedance spectroscopy for the early diagnosis of mycobacterium tuberculosis using CFP10: ESAT6 protein detection. Front. Sens. (Lausanne).

[42] Gou D., Xie G., Li Y., Zhang X., Chen H. (2018). Voltammetric immunoassay for mycobacterium tuberculosis secretory protein MPT64 based on a synergistic amplification strategy using rolling circle amplification and a gold electrode modified with graphene oxide, Fe(3)O(4) and Pt nanoparticles. Mikrochim. Acta.

[43] Chutichetpong P., Cheeveewattanagul N., Srilohasin P., Rijiravanich P., Chaiprasert A., Surareungchai W. (2018). Rapid screening drug susceptibility test in tuberculosis using sandwich electrochemical immunosensor. Anal. Chim. Acta.

[44] Rotake D.R., Anjankar S.C., Singh S.G. (2025). Multi-technique-based electrochemical sensing of lipoarabinomannan (LAM) antigen as a biomarker for early-stage tuberculosis diagnosis. Nanotechnology.

[45] Gopinath S.C.B., Perumal V., Kumaresan R., Lakshmipriya T., Rajintraprasad H., Rao B.S. (2016). Nanogapped impedimetric immunosensor for the detection of 16 kDa heat shock protein against mycobacterium tuberculosis. Microchim. Acta.

[46] Pornprom T., Phusi N., Thongdee P., Pakamwong B., Sangswan J., Kamsri P. (2024). Toward the early diagnosis of tuberculosis: a gold particle-decorated graphene-modified paper-based electrochemical biosensor for Hsp16.3 detection. Talanta.

[47] Wang L., Leng C., Tang S., Lei J., Ju H. (2012). Enzyme-free signal amplification for electrochemical detection of mycobacterium lipoarabinomannan antibody on a disposable chip. Biosens. Bioelectron..

[48] Holzinger M., Le Goff A., Cosnier S. (2014). Nanomaterials for biosensing applications: a review. Front. Chem..

[49] Holzinger M., Buzzetti P.H.M., Cosnier S. (2021). Polymers and nano-objects, a rational combination for developing health monitoring biosensors. Sens. Actuators, B.

[50] Munonde T.S., Nomngongo P.N. (2020). Nanocomposites for electrochemical sensors and their applications on the detection of trace metals in environmental water samples. Sensors.

[51] Bahrami A., Talib Z.A., Shahriari E., Yunus W.M.M., Kasim A., Behzad K. (2012). Characterization of electrosynthesized conjugated polymer-carbon nanotube composite: optical nonlinearity and electrical property. Int. J. Mol. Sci..

[52] Sang S., Wang Y., Feng Q., Wei Y., Ji J., Zhang W. (2016). Progress of new label-free techniques for biosensors: a review. Crit. Rev. Biotechnol..

[53] Raja A., Ranganathan U.D., Bethunaickan R. (2008). Improved diagnosis of pulmonary tuberculosis by detection of antibodies against multiple mycobacterium tuberculosis antigens. Diagn. Microbiol. Infect. Dis..

[54] Broger T., Marx F.M., Theron G., Marais B.J., Nicol M.P., Kerkhoff A.D. (2024). Diagnostic yield as an important metric for the evaluation of novel tuberculosis tests: rationale and guidance for future research. Lancet Glob. Health.

[55] Organization W.H.O. (2022).

